# “Feeding the Rhythm”—Effects of Food and Nutrients on Daily Cortisol Secretion: From Molecular Mechanisms to Clinical Impact

**DOI:** 10.3390/ijms262211230

**Published:** 2025-11-20

**Authors:** Rosa Maria Paragliola, Marco Marchetti, Costanza Montagna, Salvatore Maria Corsello, Gianfranco Peluso

**Affiliations:** Departmental Faculty of Medicine, Unicamillus-Saint Camillus International University of Health Sciences, 00131 Rome, Italy; marco.marchetti@unicamillus.org (M.M.); costanza.montagna@unicamillus.org (C.M.); salvatoremaria.corsello@unicamillus.org (S.M.C.); gianfranco.peluso@unicamillus.org (G.P.)

**Keywords:** cortisol, daily rhythm disruption, nutrients, clock genes, chronotherapy, metabolic syndrome

## Abstract

Daily rhythms define physical, mental, and behavioral changes that the body experiences over a 24 h cycle. The light–dark cycle plays a crucial role in regulating daily rhythms, but other factors such as food intake, stress, and physical activity also affect them. Cortisol secretion exhibits one of the largest endocrine amplitudes, with an early morning peak and late-evening nadir driven by the suprachiasmatic nucleus and hypothalamus–pituitary–adrenal axis, representing the most robust endocrine output of the circadian system. Beyond photic cues, feeding is a potent non-photic zeitgeber that entrains peripheral oscillators and dynamically shapes cortisol secretion. This narrative review aims to explore the effect of feeding in modulating cortisol secretion. The misalignment of the daily cortisol-secretion rhythm, with blunted cortisol awakening response and elevated evening levels, leads to metabolic syndrome, psychiatric disorders, shift work, and jet lag. In endogenous hypercortisolism, the loss of rhythmicity rather than absolute exposure best predicts risk. Therefore, we discuss practical nutritional tools as opportunities to partially restore rhythmic hypothalamus–pituitary–adrenal axis physiology.

## 1. Introduction

A daily rhythm is a periodic pattern where the light–dark cycles synchronize biological functions in relationship to the environment. The term was originally coined by Halberg to indicate the near-24 h endogenous oscillations of biological processes in organisms associated with the earth’s daily rotation cycle [1].

The master clock is represented by the suprachiasmatic nucleus (SCN) of the hypothalamus, which synchronizes 24 h rhythms in other areas and tissues, including other brain regions and peripheral tissues, such as the gastrointestinal tract, liver, muscle, adipose tissue, and cardiovascular tissue [2]. Therefore, the clock system is able to induce sleep and other related anabolic functions at night, while during wakefulness it regulates catabolic functions such as food intake and physical activity [2]. Several hormones (including cortisol, growth hormone, prolactin, melatonin, leptin, ghrelin, insulin, and testosterone) are secreted in a daily manner. Among those, cortisol secretion stands out for its pronounced daily oscillation, driven by SCN-mediated signals to the hypothalamus–pituitary–adrenal (HPA) axis. While physiological secretion is characterized by a predictable peak in the early morning and a nadir at night [3], the cortisol profile is dynamically modulated by behavioral cues—most notably feeding patterns—along with nutritional status and acute stressors, reflecting the complex interplay between central clock mechanisms and peripheral regulatory inputs [4].

The misalignment between central and peripheral clocks has profound metabolic consequences, and among hormonal rhythms, the cortisol cycle appears particularly vulnerable. In animal models, it has been demonstrated that clock-mutant mice, with preserved rhythmicity in the SCN and pineal gland, but with misaligned peripheral clock gene expression, present with altered glucose metabolism and altered expression of molecular determinants of metabolic homeostasis in liver and skeletal muscle [5]. In healthy humans, daily rhythm disruption can blunt or shift the normal cortisol profile, predisposing them to insulin resistance, adiposity, and dyslipidemia. In patients with established metabolic disease, these alterations are even more detrimental: impaired cortisol rhythmicity exacerbates metabolic dysfunction, while the underlying pathology further disrupts HPA axis regulation. This creates a vicious cycle in which misaligned cortisol secretion and metabolic disease perpetuate and reinforce each other.

Nutrients and overall dietary patterns play a significant role in modulating cortisol secretion, interacting with both central and peripheral components of the secreting system. The purpose of this narrative review is to analyze the effects of food intake and specific nutrients on cortisol secretion, and to discuss their potential clinical implications in specific conditions characterized by disrupted cortisol rhythmicity, such as Cushing’s syndrome (CS), metabolic syndrome (MetS), shift work, and jet lag. The literature was identified through a non-systematic search performed in PubMed, without a lower date limit, and updated to August 2025. The main keywords included “cortisol”, “circadian rhythm”, “chrononutrition”, “meal timing”, “feeding”, “Cushing syndrome”, “metabolic syndrome”, “shift work”, “jet lag”, and “clock genes”. We included original experimental and clinical studies, systematic reviews, and meta-analyses evaluating the relationship between food intake, nutrients, or feeding schedules and cortisol secretion or rhythmicity. Given the aim of our paper, preclinical models were only briefly discussed in the Section 2 to provide mechanistic background. The subsequent sections particularly focus on human data to ensure consistency and improve the interpretability of the clinical evidence.

## 2. Cortisol Daily Rhythm: Physiological Mechanisms

### 2.1. Neuroendocrine Regulation of Cortisol Secretion

Circadian (Latin “circa diem” meaning “about a day”) clocks are natural timekeepers that enable organisms to regulate their behavior and physiology with the time of the day. The rhythmic physiological functions, such as sleep/wake and feeding/fasting cycles, are coordinated by pacemaker neurons in the hypothalamic SCN. The central clock represents the master clock and drives the peripheral clocks’ oscillation in a synchronized way. These neurons synchronize cell-autonomous peripheral clocks throughout nearly all cells of our body. These clocks regulate locally rhythmic gene expression cycles of approximately 24 h, influencing physiological functions including hormone secretion [6,7,8]. At the molecular level, the clock mechanism in mammals is a negative transcription feedback loop coordinated by the heterodimeric transcriptional activator Circadian Locomotor Output Cycles Kaput/Brain and Muscle ARNT-like 1 (CLOCK/BMAL1) and its repressor complex Period (PER) and Cryptochrome (CRY) proteins. Throughout the day, CLOCK and BMAL1 proteins activate the transcription of the *PER* and *CRY* genes, whose protein products, PER and CRY proteins, inhibit and repress *CLOCK* and *BMAL1* and consequently their transcription during the night. Thus, clock genes and their protein products oscillate to generate daily rhythms [7,8].

**Figure 1:**
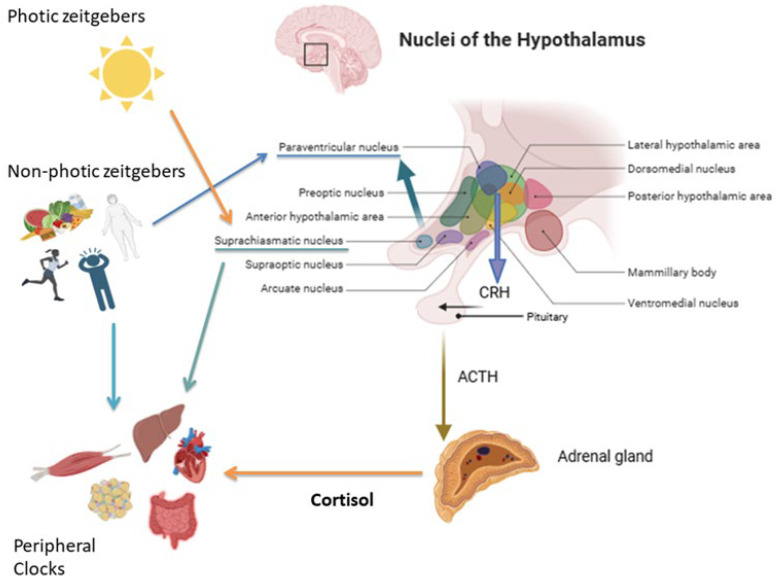
Regulation of the hypothalamic–pituitary–adrenal (HPA) axis by photic and non-photic zeitgebers. Light is the main photic cue, whereas non-photic zeitgebers include food intake, nutritional status, stress, and physical activity. Hypothalamic nuclei integrate these signals to regulate cortisol secretion via CRH and ACTH release. Cortisol, in turn, synchronizes peripheral clocks in key metabolic and physiological tissues, including the liver, skeletal muscle, gastrointestinal system, cardiovascular system, and adipose tissue, which are also directly modulated by non-photic cues.

Since the identification of central and peripheral clocks and their role in regulating biological daily rhythms, the role of glucocorticoids (GCs) has emerged for synchronizing peripheral oscillators with the central pacemaker (Figure 1).

Cortisol secretion shows a strong daily rhythm, reaching the highest level in the early morning and the lowest around midnight. The highest pulse of cortisol release occurs around wake-up time and it is defined as the “cortisol awakening response” (CAR) [9].

At the hormone level, the secretion of cortisol is controlled by the HPA axis, which is under the control of the SCN via arginine-vasopressin (AVP) projection from the SCN to the paraventricular nucleus (PVN) [10].

The “zeitgebers” (German “time givers”) are external elements that interfere with the activity of biological clocks. Light is considered the “dominant” photic zeitgeber, while other stimuli such as feeding, physical activity, temperature, and social schedules are non-photic zeitgebers that particularly influence peripheral clocks [11].

Light exposures and other non-photic cues (feeding, stress, and physical activity) activate signaling cascades in the SCN. These in turn modulate the release of corticotropin-releasing hormone (CRH) in the PVN and adrenocorticotropic hormone (ACTH) in the pituitary gland, controlling adrenal cortisol secretion [6,9]. In the adrenal gland, binding of ACTH by melanocortin 2 receptors stimulates GCs production and release. Interestingly, even if HPA axis activity is regulated by the master clock, ACTH is secreted in a lower amplitude manner compared to cortisol [12]. Therefore, the cortisol secretion rhythm is also regulated in an ACTH-independent manner, through the innervation from the autonomous nervous system via the splanchnic nerve [9].

Cortisol binds and activates the GCs receptor (GR) in peripheral tissues and regulates local circadian clocks, allowing for tissue-specific regulation. Using hormone fluctuation levels and tissue-specific mechanisms, cortisol shapes physiological responses during the day, functioning as a messenger from the central clock to peripheral tissues and synchronizing local clocks and physiological processes throughout the body [2,13,14].

On the other hand, peripheral tissues modulate their sensitivity to cortisol’s daily rhythm via the core circadian regulator CLOCK protein. It has been demonstrated that the CLOCK protein modifies the GR by acetylation, reducing its activity in the morning, when cortisol is high, and increasing sensitivity at night, when cortisol is low [15,16].

Also, GR activation by daily cortisol pulses leads to rhythmic gene-specific transcription [17]. Among these genes, the circadian regulator *PER1*, is overexpressed at low GC concentrations (similar to the night-time concentration of human cortisol) in human and mouse pituitary cells, indicating a dose-specific GC response, which allows for precise control of gene expression in target tissues [18].

All these regulatory mechanisms prove that peripheral clocks can be synchronized with tissue-specific signals, while the SCN is driven by the light–dark cycle [9]. Interestingly, the SCN is not regulated by the synchronizing activity of GCs, probably because of the lack of GR expression in this region. However, GR expression has been recently demonstrated in astrocytes of the adult SCN, suggesting a possible GCs effect on the SCN more evident than previously supposed [19].

However, the whole system remains sensitive to environmental and hormonal cues that can derail central–peripheral synchrony and blunt endocrine rhythms. External stressors such as changed light exposure or prenatal GC exposure can disrupt clock gene expression and induce the loss of rhythmicity in both central and peripheral clocks [2,20]. Indeed, night-shift work diverts the cortisol rhythm, causing metabolic and cardiovascular disorders [20].

In conclusion, it is important to underline that light exposure represents the primary zeitgeber for the central SNC, while feeding behavior acts as a non-photic zeitgeber with weaker entrainment power on the SCN but a predominant influence on peripheral clocks. Time-restricted feeding can induce phase shifts of up to 12 h in peripheral clocks without significantly altering the central pacemaker, thereby decoupling metabolic rhythms from the SCN’ light-driven cycle [21]. Therefore, feeding alone is insufficient to entrain the central SCN clock, even when light, which represents its primary zeitgeber, is absent. However, meal timing acts as a powerful synchronizing cue for peripheral oscillators, capable of overriding signals from the light-entrained SCN [22]. Several other studies have demonstrated that food intake can reset the phase of peripheral clocks without influencing the SCN [23,24]. Consequently, in cortisol regulation, which depends on both central and peripheral mechanisms, light exposure remains the dominant signal regulating cortisol secretion, while meal timing modulates peripheral and metabolic components, influencing the amplitude and timing of postprandial cortisol excursions.

### 2.2. Role of Nutrients on Clock Genes Expression

Nutrients act not only as energy substrates but also as signaling molecules able to module circadian rhythmicity through direct and indirect effects on clock gene expression. Therefore, feeding is included in the non-photic time stimuli synchronizing the endogenous clock [25], interacting with the SCN through mediators such as orexin neurons, ghrelin, and leptin receptors [13]. Macronutrient composition, meal timing, and specific bioactive compounds can influence the transcriptional activity of core clock genes, thereby affecting downstream metabolic and endocrine pathways, as well as the action of GRs [26].

The phase of the central clock is synchronized to light/dark cycles. In this case, signal input derives from the optic nerve. In mice, the disruption of standard light–darkness cycles to a continuous light cycle, caused increased food intake and, even in the presence of similar caloric intake and total motor activity with controls, led to excess weight gain [27]. On the other hand, the synchronization of peripheral clocks is obtained not only by the hypothalamic central clock through the peripheral nervous system, but also through other factors, including the nutrients absorbed after food consumption. Therefore, in the absence of light-sensing the other biological clocks throughout the body, it is regulated by feeding cycles and this phenomenon is called “food entrainment” [28].

The time of feeding can influence peripheral clocks, while the central clock appears to be “resistant” to a transient shift in feeding time. Eating against the central clock causes internal desynchronization. In particular, among peripheral clocks, the hepatic clock is particularly sensitive to feeding time [21]. The metabolic effects of these habits can be deleterious. Mouse models fed during the resting phase, which showed altered peripheral clock genes expression, gained weight, developed abdominal obesity and metabolic alterations, and reduced physical activities [29]. Consistently with mouse models, similar patterns are seen in humans, in whom making breakfast the largest meal predicted greater body mass index (BMI) reduction [30] than lunch or dinner [31]. Furthermore, a delay in meal timing significantly phase-shifted peripheral rhythms. A 5 h delay in meal timings (breakfast, lunch, and dinner) altered adipose tissue *PER2* expression by about one hour and plasma glucose rhythms by nearly six hours, with a concomitant reduction in average glucose levels [16].

Diet composition has been reported to affect biological clocks in animal models. A high-fat diet in mice under constant darkness prolongs daily locomotor rhythms [32]. Although the mechanism is not clear, a fat diet alters feeding behavior, hepatic transcript oscillations (PPARγ-dependent), and lipid metabolism (PPARδ-mediated lipogenesis and PPARα activation). The increase in blood sugar levels downregulates Per1 and Per2 expression in mice fibroblasts [33], representing a model cell system of the peripheral clock, supporting glucose as a key molecule capable of directly resetting peripheral clocks.

On the other hand, genetic susceptibility can influence the chronotype, as demonstrated in several studies involving humans [34,35]. This genetic perspective adds to the evidence linking the evening chronotype with adverse metabolic outcomes. A recent study, the “European Prospective Investigation into Cancer and Nutrition (EPIC) chronodiet” study, comprised 3183 subjects with information on diet and twelve genetic variants of six genes (*PER1, PER2, PER3, CRY1, NR1D1*, and *CLOCK*). In evening or late chronotypes, a misalignment between nutrient intake and biological rhythm has been found, with breakfast providing a smaller proportion of daily energy and macronutrients—particularly proteins and carbohydrates—compared with early or intermediate chronotypes. A genetic risk score for evening chronotype and obesity (variants of *CLOCK, PER3*, and *CRY1*) has been found, suggesting that genetic predisposition can shape chrononutrition patterns [36].

### 2.3. Physical Exercise: The Timing of Physical Activity in the Regulation of HPA Axis

Physical activity is well known to highly impact the neuroendocrine system and hormonal secretion [37]. Exercise represents a non-photic zeitgeber able to influence both the phase and the amplitude of glucocorticoid rhythmicity, thereby affecting daily metabolic homeostasis. Most of the available data on exercise-induced hormonal modulation derives from studies performed on athletes, where chronic training elicits well-characterized adaptations of the HPA axis. In this setting, the correlation between physical exercise and cortisol secretion is complex, because intensity, duration, and timing of exercise profoundly influence cortisol secretion and the balance between catabolic and anabolic processes [38]. In particular, the timing of physical activity represents a critical determinant of cortisol secretion and HPA axis activation.

Human studies have demonstrated that morning exercise, performed soon after awakening, induces a more pronounced acute rise in cortisol compared to afternoon or evening sessions. The evaluation of GH and cortisol secretion in response to exercise at different times of the day, showed that while GH secretion was unaffected by exercise timing, cortisol release is regulated by a circadian modulation [39]. Exercise induced a significant rise in serum cortisol at all time points (07:00, 19:00, and 24:00 h), but the magnitude of the increase was greatest at midnight and lowest in the evening. Baseline and peak cortisol concentrations were highest in the morning, according to the physiological morning cortisol acrophase. Moreover, a transient post-exercise suppression of cortisol occurred only after the midnight session, suggesting altered feedback sensitivity at that time [39].

Morning aerobic exercise—particularly when performed regularly—is associated with reduced CAR and improved sleep architecture. In contrast, high-intensity or prolonged evening exercise can elevate nocturnal cortisol, increase core body temperature, and delay melatonin onset, thereby impairing sleep quality [40]. Among athletes, frequent evening or high-intensity sessions are linked to higher nocturnal and post-awakening cortisol levels, and poorer sleep quality, especially during overtraining phases [40]. Conversely, moderate exercise at appropriate times appears to downregulate HPA axis activity, restoring normal cortisol rhythmicity. Furthermore, the timing of exercise may influence its metabolic efficacy, with morning sessions exerting favorable effects on cardiometabolic regulation. In MetS, morning aerobic training improves insulin sensitivity and reduces systolic blood pressure more effectively than afternoon exercise [41].

From a physiological point of view, exercise acutely activates the sympathetic adrenal system and stimulates CRH and ACTH release, leading to cortisol secretion with subsequent catabolic and recovery phases. The timing of this activation modulates both the recovery window and post-exercise phenomena, influencing peripheral clock activity [42] in tissues such as skeletal muscle and adipose tissue. Consequently, the integration of exercise timing into chrononutritional strategies may be helpful in restoring cortisol rhythm regulation.

## 3. Nutritional Regulation of Cortisol Secretion

### 3.1. Effects of Meal Timing and Frequency on Cortisol Secretion (Chrononutrition)

It is important to underline the difference between the terms “Chrononutrition” and “Chronotherapy”. Chrononutrition explores the relation between the timing of nutrient assumption and some hormone secretion. Chrononutrition influences the amplitude and phase of cortisol secretion, particularly by modulating postprandial metabolic signals that interact with the HPA axis. On the other hand, the term chronotherapy is better related to the timing of administration or assumption of some drugs, according to circadian rhythms of hormone secretion, metabolism, and tissue sensitivity. In the case of cortisol regulation, chronotherapy aims to restore physiological cortisol rhythmicity.

The cortisol increase in response to food intake was first described in the 1970s and has been confirmed in other studies both in normal-weight and in obese subjects [43]. The timing of food intake represents a key factor for daily rhythm physiology, with cortisol secretion dynamically adapting to meal timing. However, eating itself is not only a metabolic signal but also a multifaceted behavior shaped by physiological needs, habits, cultural context, and, in many cases, religious prescriptions. Recent studies show that meal timing is not strictly related to the three- or five-time meal pattern, but on the contrary, calories are assumed to be spread over a wide timespan [44]. Different eating habits and patterns, as well as meal timing, might influence body composition, metabolism, hormone secretion, and health [45]. For example, having a late dinner or, conversely, having an early breakfast and lunch, could deeply interfere with health status. Night-time meals, even small snacks, are linked to weight gain and poorer health outcomes, a trend particularly evident among night-shift workers and adolescents who stay up late and eat at night [46]. After a meal, cortisol levels rise within 30 min, peak at around 1 h, and return to baseline in about 2 h [47], closely reflecting meal timing [48]. Fasting can alter this pattern. People who fast have two cortisol peaks during sunrise, while people who do not fast have just one. These findings suggest that during a fasting period, which spans from sunrise to sunset, fasting-induced cortisol secretion helps align the peripheral and central clocks and prevents misalignment. Ramadan, a holy month in the Muslim tradition characterized by abstention from food during daylight hours, offers a natural model of this phenomenon. During Ramadan, some studies describe a biphasic cortisol rhythm aligned with the first post-fast meal [48], while others report a significant decrease in morning cortisol either throughout the month [49] or only towards its end [50]. Ramadan could be considered as a “fasting strategy”. However, analyzing some non-Ramadan restricted feeding studies, different findings have been reported, such as a decrease in evening cortisol and potentially an increase in this hormone upon waking. In time-restricted eating (TRE), to have breakfast and skip dinner, or fast during the day, determines different cortisol secretion. Skipping breakfast is associated with a significant reduction in morning cortisol levels, a pattern that may indicate HPA axis dysfunction and is linked to cardiometabolic deterioration [51]. Overall, this meal pattern tends to shift the cortisol curve to the right, leading to higher levels later in the day. In fact, skipping breakfast increased midday cortisol levels, regardless of the calorie restriction applied [45]. Women who skipped breakfast had significantly higher cortisol levels [51], a pattern that could be due to the delayed action on the HPA axis, which also affects the next meal. In other words, the postprandial rise in cortisol is superimposed on the cortisol already elevated due to sympathetic activation of the HPA axis. Conversely, subjects accustomed to skipping dinner showed significantly lower cortisol levels [52]. This is likely due to the anticipatory cortisol response, according to which cortisol rises in anticipation of a meal. TRE also causes an increase in the amplitude of cortisol secretion rhythm only if the evening meal is omitted. In light of these considerations, choosing TRE can have significant effects on productivity. Skipping dinner may therefore support increased morning activity and better nighttime sleep, as lower daytime cortisol levels can improve sleep quality [52]. These effects seem to occur independently of total calorie intake [45]. Caloric restriction could be a stressor factor, and just for that, could lead to cortisol rise [53]. In a study comparing TRE with a conventional diet, with the same caloric deficit of approximately 25%, the results showed a decrease in cortisol following TRE, probably because fasting alters the normal daily secretion rhythm [54].

### 3.2. Nutrient Composition and HPA Axis Activity

The HPA axis regulates GC production and is strictly related to meal composition. Clinical intervention with ascorbic acid (vitamin C) showed a reduction in cortisol reactivity in healthy adults. The supplementation of Omega 3 fatty acids is associated with improvements in cortisol levels, while polyphenol-rich foods such as chocolate or pomegranate, reported a reduction. On the other hand, a small study (just 3 days and 12 participants) demonstrated that a high glycemic diet is associated with an increase in cortisol secretion [55]. Omega-3 polyunsaturated fatty acids, particularly EPA and DHA, can regulate the HPA axis by reducing cortisol production, while 7.2 g of fish oil per day for three weeks was sufficient to induce a reduced stress response in healthy men [56].

Potassium supplementation could possibly also elevate cortisol concentration [57]. The mechanism by which those nutrients influence cortisol secretion is not yet clear; it is probable that the influence is mediated by a modulation of pro-inflammatory response to hypotonic activation [58]. The use of multi-strain probiotic supplements demonstrated an improvement in free urinary cortisol in healthy people [59].

It is important to underline that a ketogenic diet (KD) could play a different role in influencing the HPA axis. A KD is approximately a dietary pattern with 5% of carbohydrates, 15% of protein, and 70% of lipids which stimulate the production of keto bodies.

Several anthropometric and metabolic parameters, including BMI and visceral adiposity, are associated with alterations in cortisol secretion. According to Polito [60], very low-calorie KD (VLCKD) includes some important changes in cortisol secretion, through improvements in BMI, total cholesterol, and adiponectin. Serum cortisol levels decrease following the decrease in cortisol-binding proteins in VLCKD. Conversely, as reported by Guarnotta [61], it is possible to hypothesize that VLCKD, by reducing visceral fat, could lead to a lower expression of type 1 11-β-hydroxysteroid dehydrogenase (11β-HSD1), which catalyzes the conversion of corticosterone to cortisol and enhances the inactivation of cortisol in cortisone, increasing 11β-HSD2 expression.

It is important to consider that diet is a stress factor for the individual. Several studies from the last decade have demonstrated that the KD stimulates the production of metabolic regulatory hormones, including cortisol [62], as well as that the relative depletion of dietary carbohydrates, inducing ketosis, acutely and chronically activates the HPA axis [63]. On the contrary, in human studies, checking salivary cortisol levels during a ketogenic diet with a very-low calorie amount, the authors found that VLCKD had a short-term positive effect on the HPA axis, and cortisol level significantly decreased, despite, that normally, VLCKD was related to hyperactivation of the HPA axis [60]. Finally, a specific consideration regards carbohydrates and their effect on cortisol secretion. In fact, even if cortisol is a counter-regulatory hormone and its secretion is stimulated by hypoglycemia, an evening high-glycaemic meal often raises cortisol via neuroendocrine and counter-regulatory mechanisms. Firstly, insulin sensitivity and glucose tolerance later in the day are reduced and evening high-glycemic load causes a wider glycaemic/insulinaemic excursion. Rapid post-meal hyperglycemia can cause reactive hypoglycemia, which in turn activates a counter-regulatory response (increased epinephrine, glucagon, and obviously cortisol secretion) and then a reactive hypercortisolemia [64].

It is also important to underline that sex differences may substantially influence metabolic regulation and hormonal responses to feeding. Recent evidence highlights distinct post-prandial profiles between men and women in gastric emptying rates, which are slower in women than men with type 2 diabetes [65]. Furthermore, post-prandial hyperglucagonaemia is more prominent in males than in females after a nutrient load in diabetes [66], while healthy young women present with greater GLP-1 response to intraduodenal glucose than men [67]. These differences may contribute to sex-specific patterns in cortisol dynamics and metabolic adaptation to dietary interventions.

### 3.3. Dietary Pattern and Daily Cortisol Secretion

GCs secretion and cortisol levels are strictly related to the quality of diet, and meal timing could also influence adrenal and extra-adrenal activity. The association between meal timing and blood cortisol concentration is known and involves adrenal and extra-adrenal regulation [59]. For example, having a late dinner might lead to reduced free fatty acid, delayed triglyceride peak, and increased cortisol level [60].

It is important to underline that GC release and meal composition have a bidirectional interaction. The diet can influence cortisol and GC secretion, but, on the other hand, lower numbers of a cortisol peak can increase the consumption of a specific dietary pattern characterized by high caloric intake through high amounts of simple sugar and saturated fatty acids [61]. The Western diet alimentary pattern, characterized by a minimum fiber intake but a high amount of salt, saturated fatty acids, and simple sugar, is strictly related to an increase in GCs, cortisol in particular, and might be related to greater oxidative stress [62]. Also, an isocaloric-rich protein diet might increase lean mass, testosterone, ghrelin, and growth hormone, but also cortisol level [63].

In an observational study, a dietary pattern characterized by high intake of saturated fatty acids but low intake of fruits and vegetables can alter daytime cortisol levels. In this regard, it is important to underline that the acid-base balance can influence adrenal function and, consequently, cortisol secretion. The reduction in bicarbonate levels could increase glutaminase activity and increase cortisol production [68]. Some dietary patterns, rich in protein and poor in fruits and vegetables, can determine metabolic acidosis and might be related to high cortisol concentration [69]. In this regard, it is important to underline how some specific dietary patterns, such as the Mediterranean one, could ameliorate cortisol levels. Following a healthy and balanced diet, with a high amount of vegetables, fruit, and whole grains, but, at the same time, a low amount of simple sugar, saturated fatty acids, and salt, could decrease the urinary cortisol level [70].

### 3.4. Role of Gut Microbiota in Modulating Cortisol Rhythms

The composition of the gut microbiota and its activity is strictly related to the HPA axis, and cortisol level could affect the gut microbiota–brain axis in several ways. For example, it is known that cortisol receptors are present in epithelial, immune, and enteroendocrine cells, and cortisol can also determine gut composition, influencing intestinal permeability and nutrient availability [71]. Even if according to Shibata [72], the changes in gut microbiota composition in childhood experiments are mostly due to the relaxing environments, some authors found deep relationships. For example, Ravenda found that, in some young students, the high concentration of Lactobacillus and Bifidobacterium is related to lower levels of salivary cortisol if compared with other students with a microbiota with predominant Bacteroides [73]. On the other hand, other authors showed that gut microbiota diversity is associated with post-stressor cortisol concentration in a study on 2.5 aged children [74].

The interaction between gut microbiota and the HPA axis is often stress-mediated. Some neurons in the hypothalamic paraventricular nucleus synthesize and secrete corticotropin-releasing hormones and antidiuretic hormones in response to stress. All those compounds facilitate adrenocorticotropic hormone release, and consequently, cortisol secretion. On the other hand, cortisol influences the activity of the hippocampus and pituitary gland with negative feedback [13]. Chronic stress is associated with high cortisol levels, and both are associated with higher gut permeability, leading to an inflammatory status. The intestinal barrier, made more permeable by cortisol, allows passage from the intestinal lumen to the bloodstream, increasing the parameters of systemic inflammation [75]. Also, fear is associated with an increase in cortisol levels and peculiar production of some gut metabolites. In a human model, the supplementation of short-chain fatty acids to the colon was enough to protect from an increase in cortisol induced by fear and psychological stress [76].

## 4. Disruption of Cortisol Secretion Rhythm in Human Diseases

The daily rhythm of cortisol secretion is a finely regulated process essential for maintaining homeostasis and coordinating metabolic, immune, and neuroendocrine functions. Disruption of this rhythm, whether due to intrinsic pathological mechanisms or external environmental influences, is increasingly recognized as a critical factor contributing to disease onset and progression. In CS, the loss of daily rhythmicity reflects autonomous and excessive cortisol production, with profound systemic consequences. Similarly, in psychiatric disorders, the dysregulation of cortisol rhythmicity is closely linked to altered stress responsiveness, impaired emotional regulation, and increased vulnerability to mood and anxiety disturbances. In metabolic disorders such as diabetes and obesity, subtle but significant alterations in cortisol secretion and its diurnal profile exacerbate insulin resistance, adiposity, and cardiometabolic risk. Moreover, conditions of daily rhythm misalignment, such as jet lag or shift work, demonstrate how environmental perturbations of the HPA axis can also cause long-term clinical effects. Understanding these mechanisms may be useful in clarifying if supportive nutritional strategies could help in these clinical scenarios. Salivary cortisol assessment, a non-invasive and easy to perform tool, is particularly useful in demonstrating these alterations.

Other quantitative approaches to assess cortisol rhythmicity include the CAR, the diurnal slope, and the area-under-the-curve (AUC) over 24 h. The CAR quantifies the rapid increase in cortisol secretion within the first 30–45 min after waking, reflecting the reactivity and integrity of the HPA axis [77], while the diurnal slope describes the rate of cortisol decline from morning to evening values [78]. The AUC over a 24 h period integrates total cortisol exposure and the distribution of secretion across the day [79]. Flattening of the diurnal slope or blunted CAR values are associated with metabolic, cardiovascular, and neuropsychiatric diseases, even in the absence of overt hypercortisolism [77]. These findings support the concept that a loss of rhythmicity, rather than absolute cortisol exposure, represents a key marker of HPA axis dysregulation and its consequences.

### 4.1. Endogenous Cushing’s Syndrome

**Figure 2:**
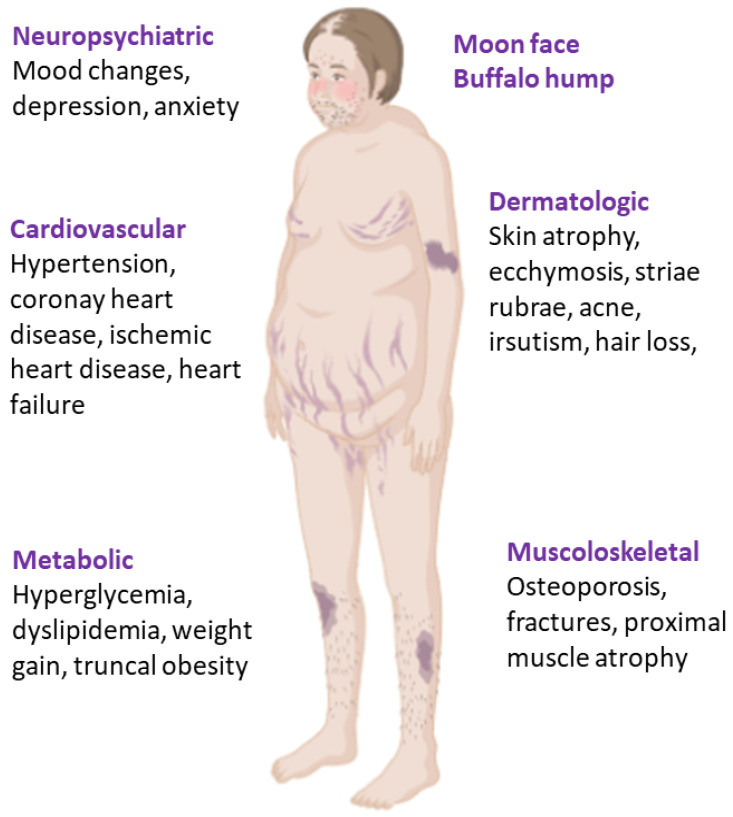
Clinical features of Cushing syndrome.

Cushing syndrome is a severe disorder caused by chronic GC excess, most often iatrogenic, or from ACTH-dependent/independent endogenous overproduction. Inappropriate cortisol secretion leads to severe multisystem morbidity for its cardiometabolic complications and increased mortality, often due to pulmonary embolism, infection, myocardial infarction, and cerebrovascular accidents [80,81]. Furthermore, even after remission, the risk of mortality is higher compared with the general population and patients can experience long-term side effects of the previous chronic and prolonged exposure to inappropriate endogenous GC levels [82], probably mediated by hypercortisolism-induced long-lasting epigenetic changes [83]. The main clinical features associated with CS have been reported in Figure 2.

The diagnosis, which is often very challenging, relies as a first step on tests such as 24 h urinary cortisol, late-night salivary cortisol, and a low-dose dexamethasone suppression test, which detect daily cortisol overproduction, loss of daily rhythm, and impaired feedback inhibition, respectively [84]. In particular, the disruption of cortisol’s daily rhythm is considered a key factor both in the pathophysiology of CS and as a cause of clinical complications, which are largely attributed to the failure of plasma cortisol levels to physiologically drop in the late evening and at night. Therefore, endogenous CS, being the most frequent etiology due to an ACTH-secreting pituitary adenoma (Cushing disease, CD), represents the most illustrative model of daily rhythm disruption [85]. Notably, disturbances in the 24 h cortisol rhythm—especially loss of the late-night nadir—may act as an early, preclinical signal of hypercortisolism in both adrenocortical and pituitary tumors [86]. Clinically, this is valuable, as late-night salivary cortisol (or midnight serum cortisol) is often the first abnormality, enabling earlier detection of mild or intermittent disease and providing sensitive clinical parameters both for the diagnosis and for follow-up of apparently “cured” patients, as an early biochemical marker of persistent or recurrent disease.

From a physio-pathological point of view, the inappropriate cortisol secretion observed in CS acts as a pathological signal modulator of the clock gene system, leading to abnormal expression of clock genes and daily rhythm disturbance in peripheral tissues. The physiological HPA axis’ negative feedback induced by GCs is absent in endogenous CS, as shown by its failure to suppress serum cortisol below ~1.8 µg/dL after the low-dose (e.g., 1 mg overnight) dexamethasone suppression test [87]. In CD there is tonic autonomous ACTH secretion from the pituitary tumor, but the feedback resistance is often partial (as demonstrated by the cortisol suppression after high dose—8 mg dexamethasone suppression test in most patients). Despite this partial suppression, chronic hypercortisolism downregulates hypothalamic CRH output and uncouples pituitary ACTH from its normal daily drive, producing a flattened diurnal ACTH/cortisol profile with elevated late-night levels [88]. This phenomenon in turn may contribute to dysregulation of the peripheral clock system, causing the metabolic alterations and the clinical features of CS [13].

Unlike healthy subjects who display clear daily oscillations of multiple clock genes, patients with CD lose this rhythmicity in parallel with the abolition of cortisol daily secretion, with CRY2 emerging as a potential marker of rhythm disruption [89]. Consistent with the above-mentioned study, a recent multicenter prospective trial confirmed that in CS the daily rhythmicity of both immune cell oscillations and core clock gene expression (*CLOCK*, *PER1–3*, and *TIMELESS*) is profoundly blunted or lost, with only partial recovery after remission, underscoring daily disruption—rather than absolute cortisol levels—as the key hallmark of chronic GC excess [85].

For ACTH-independent CS, studies exploring clock genes expression in human tumors found a dysregulated clock gene expression compared with the normal adjacent adrenal tissue, with a different pattern between benign and malignant tumors [90]. In patients with adrenal CS (ACTH-independent), increased cortisol secretion results from both increased basal secretion and increased pulse frequency [91] and a partial persistence of cortisol rhythmicity has rarely been observed.

Beyond overt CS, loss of daily rhythmicity is evident in mild autonomous cortisol secretion (MACS), which represents the most common hormonal alteration reported in up to 45% of patients with adrenocortical adenomas. According to recent 24 h urine steroidomics data, evening total and free cortisol were higher in MACS, consistent with blunting of the nocturnal nadir [92]. Therefore, MACS has to be considered a state of subtle daily dysregulation rather than of simple hyperexposure and underscores the clinical value of late-evening/overnight measures and day–night urine profiling for detection and follow-up.

In the group of ACTH-independent hypercortisolism, food-dependent CS represents the most representative model linking food cues to inappropriate cortisol secretion. This condition is a rare ACTH-independent hypercortisolism in which meals trigger cortisol surges via aberrant adrenal expression of the gastric inhibitory polypeptide (GIP) receptor, most often within primary bilateral macronodular adrenal hyperplasia [93]. The effects of meals are able to override SCN-driven diurnal control; fasting morning cortisol may be relatively low while post-meal or OGTT-provoked values are inappropriately high, and midnight cortisol is elevated, with inverse diurnal rhythm. Plasma cortisol levels can increase dramatically in response to a lipid-rich meal, to a protein-rich meal, and to oral glucose, with increased plasma cortisol levels closely correlated to increases in GIP concentrations [94].

### 4.2. Metabolic Syndrome

Metabolic syndrome is a complex disorder, considered a worldwide epidemic, and defined by several pathological conditions to increase the risk of cardiovascular disease and type 2 diabetes. This condition is bidirectionally linked to a dysregulation of physiological cortisol secretion. In fact, if inappropriate cortisol secretion (both in CS and in different clinical scenarios related to the disruption of normal cortisol’s daily rhythm) is associated with an increased risk to develop MetS, on the other hand MetS itself is associated with disruption of the diurnal cortisol pattern. Hypercortisolism causes an increase in inflammation, correlated with the incidence of carotid artery plaques (as found in vascular atherosclerosis) and is associated with stroke and linked to poor post-stroke outcomes and mortality [78]. In MetS, the dysregulation of the timing of cortisol secretion—rather than large changes in absolute daily output—plays the major role. The common characteristics of MetS and the hypercortisolemic state (as in CS) suggested that a prolonged exposure to inappropriate GC secretion can cause clinical features related to MetS. Actually, hyperactivity of the HPA axis has been reported in MetS, leading to a state of “functional hypercortisolism [95]”.

Several clinical studies suggest that MetS and its components (abdominal obesity, disorders of glucose metabolism, hypertension, and dyslipidemia) are associated with flattening on diurnal cortisol secretion, with a blunted CAR and higher evening–bedtime cortisol, even without elevated 24 h cortisol secretion or mean daytime levels [96]. In the Whitehall II cohort, a flatter daytime decline and higher bedtime cortisol predicted later impaired glucose metabolism and type 2 diabetes, supporting a causal effect between daily cortisol secretion misalignment and metabolic risk [96,97]. Interestingly, hair cortisol increase, which indexes long-term exposure to GCs, is positively associated with MetS and several of its components, suggesting chronic up-shifting of baseline HPA axis activity in metabolically unhealthy states [98].

However, several studies underline that basal (single time point) cortisol measurements have inconsistent associations with MetS [99], underscoring the importance of the pattern of cortisol secretion (blunted CAR and elevated evening nadir levels). These findings have also been confirmed for the pediatric population, in which obesity is associated with lower morning and higher night salivary cortisol [100].

From a clinical point of view, a more accurate dynamic assessment of cortisol secretion is necessary (e.g., multi-sample salivary profiling over the day, focusing on late-night salivary cortisol), rather than reliance on isolated basal measurements.

The causes related to the hyperactivation of the HPA axis, with a daily HPA axis “dys-synchrony” in MetS, characterized by an attenuated morning surge and lack of nocturnal nadir, remain uncertain. They could be related to chronic stress, which is associated with increased responsiveness of HPA axis activity, and consequently, increased circulating cortisol levels [95]. Increased cortisol secretion contributes to increased fat accumulation in visceral depots. Furthermore, increased 11β-HSD1 activity in adipose tissue and liver amplifies local cortisol action in these tissues, and might be involved in the development of several features of MetS [101,102].

### 4.3. Psychiatric and Behavioral Disorders

Disruption of cortisol’s daily rhythm is a frequent neuroendocrine feature in several psychiatric disorders, including schizophrenia, bipolar disorder, and depression [103]. Several studies have shown a blunted CAR, elevated evening cortisol levels, and overall flattening of the diurnal slope. Patients affected by depression often exhibit elevated late-evening salivary cortisol and impaired suppression after dexamethasone, suggesting both loss of daily rhythmicity and altered feedback of the HPA axis [104]. Similarly, bipolar disorder and post-traumatic stress disorders are associated with a reduced CAR, demonstrating a disruption of stress-responsive cortisol secretion [105]. Genetic studies have highlighted the involvement of circadian clock genes in bipolar disorder, although findings should be validated by more robust studies. *CLOCK* and *BMAL1* variants are linked to evening chronotypes, delayed sleep, higher recurrence, and lithium response. PER variants have been reported in association to age of onset of mental disorders, treatment response, mood fluctuations, and chronotype changes, while *CRY* is associated with sleep disorders and lithium response [106].

As for MetS, there is a bidirectional relationship between inappropriate cortisol secretion and mental health. Psychiatric disorders and chronic psychological stress can activate HPA axis activity leading to chronic hypercortisolism; however, elevated cortisol levels themselves can produce detrimental effects on mental health, causing mood alterations, cognitive impairment, and psychiatric morbidity. These features are well recognized in overt CS, but interestingly, recent evidence underlined that they extend to milder forms of inappropriate cortisol secretion as well. In a group of patients with adrenal incidentalomas, those with “subclinical CS” (MACS) showed greater detrimental effects on mental health compared with those with non-hypersecreting adrenal incidentalomas [107]. On the other hand, the most severe expression of the relationship between psychiatric disorders and inappropriate cortisol secretion is the “pseudo-Cushing syndrome”, in which the dramatic loss of cortisol’s daily control can lead to a biochemical and clinical picture resembling CS. Patients with major depression or alcohol use disorders may present persistent non-neoplastic clinical and biochemical hypercortisolemia (loss of the nocturnal nadir in salivary cortisol, and abnormal results on screening tests such as urinary free cortisol or low-dose dexamethasone suppression test) [108]. The diagnosis of this condition is often challenging but crucial to plan the correct therapeutic approach [109]. In fact, unlike neoplastic hypercortisolism, the abnormalities related to the pseudo-Cushing state often normalize with remission of the psychiatric condition, underscoring the functional and reversible nature of the disorder.

### 4.4. Cortisol Daily Rhythm Disruption in Shift Workers and Chronic Jet Lag

Daily rhythm disturbances caused by conflicting synchronizing cues (e.g., shift work and recurrent jet lag) are now considered as pathogenic when recurring on a chronic basis, instead of an ordinary adaptive mechanism. In fact, the daily rhythm of shift workers rarely adapts to work changes [20], because the vast majority of them revert to ordinary daytime activities during non-work days. Regarding frequent fliers, as is well known, the condition characterized by the desynchronization of the daily rhythm is so-called “jet lag” syndrome, associated with long-haul flights across several time zones. The cause of jet lag is the persistence of the “body clock” function in the day–night rhythm of the place of departure. This syndrome causes the lack of alertness, poor sleep, irritability, stress, impaired performance in athletes, and depressed mood [110]. Both night-shift workers and frequent fliers prone to an altered daily rhythm present with increased risk for metabolic syndrome and tumors [85]. The disruption of cortisol secretion can represent a reliable marker both of jet-lag syndrome and shift-worker cortisol impaired secretion. Furthermore, the disruption of GC rhythmicity is probably implicated in the above-mentioned physical alterations. Night-shift workers displayed significantly lower cortisol levels upon waking compared to day-shift workers [20]. They also present higher self-reported stress and fatigue levels, as well as a blunted CAR [111], which reflects a diminished ability of the HPA axis to adapt to acute stressors. Interestingly, a study involving junior physicians demonstrated that those engaged in shift work had significantly higher waking salivary cortisol levels, a steeper diurnal slope, and an increased total cortisol output one year later, compared with their day-shift colleagues [112]. In this case, the chronic stress associated with shift working could be associated with a “chronic” functional hypercortisolism. Among airline pilots, salivary cortisol sampling revealed distinct cortisol daily rhythm disruptions. In fact, the CAR and nightly rhythm varied markedly between shift days and rest days, underscoring instability even within the same individuals [113]. Jet-lagged travelers also showed an important cortisol dysregulation: in one study involving transcontinental flight healthy subjects, a post-travel inversion of the physiological pattern of cortisol secretion has been observed, with higher midnight salivary cortisol and very low morning cortisol levels [114]. Based on their data, the authors assumed that after a West-to-East fly, the set-point of cortisol secretion remains still synchronized on the West time zone for at least 36 h from the return to East [114]. This process represents an extreme loss of synchronization between the internal daily rhythm and external time and can be associated with the clinical features of jet-lag syndrome.

## 5. Strategies for Cortisol Rhythm Restoration

The restoration of a physiological cortisol daily rhythm requires, in most cases, the treatment of its underlying condition. However, personalized chrononutrition as well as a specific and pharmacological approach by using specific medical therapies or supplements, could be helpful in aligning zeitgebers. These approaches can contribute to reducing risks associated with cortisol rhythm disruption. On the other hand, the relationship between cortisol and feeding is bidirectional. In fact, GC excess per se is associated with the development of obesity (and visceral fat accumulation) and their complications through different mechanisms. GCs increase the preference for palatable, high-caloric foods which predisposes overconsumption and increased weight [115]. This mechanism involves an altered expression of several neuropeptides regulating fasting/feeding homeostasis. Neuropeptide Y (NPY) expression, the strongest endogenous orexigenic neuropeptide increasing feeding [116], is induced by GCs. On the contrary, in the condition of chronic hypercortisolism, levels of leptin and adiponectin, which in turn antagonize NPY expression, are reduced, while Ghrelin levels rise, contributing to increased food intake and weight gain [9]. Therefore, nutritional prescriptions are not merely adjunctive but an integral tool to mitigate this “feed-forward loop” and to improve the restoration of rhythmic HPA axis physiology. A summary of nutritional, pharmacological, and supplemental strategies potentially useful to restore or modulate daily cortisol rhythm is reported in Table 1.

### 5.1. Personalized Nutritional Suggestions: Practical Considerations

The common thread of the different clinical conditions associated with cortisol’s daily rhythm disruption is the loss of a steep morning rise and low evening cortisol nadir. In general, avoiding late-night eating is useful in realigning peripheral clocks with the central pacemaker, avoiding evening cortisol elevations. Early window time-restricted eating and consequent early day energy distribution improves glycemic profiles and regulate clock genes markers [128]. In conditions of hypercortisolism, the GC excess amplifies post-meal metabolic stress, with hyperglycemia, hypercholesterolemia, and hypertension being frequent complications. Therefore, small, low-glycemic, lower-fat meals and avoiding late dinners can reduce meal-provoked cortisol excursion.

Available interventional suggestions in shift workers mainly indirectly derives from controlled studies on meal timing and time-restricted feeding, showing partial restoration of metabolic and hormonal rhythmicity. Other recommendations are derived from observational data and should be considered translational hypotheses rather than evidence-based clinical guidance. For shift workers, the suggestions are to avoid eating or reduce food intake between midnight and 6 a.m., using the normal day and night pattern of meal timing as much as possible, and to divide eating into three meals per 24 h period [129]. The consumption of carbohydrates at night increases sleepiness and degrades cognitive performance and should be avoided, while protein-rich options enhance satiety/alertness [117]. Therefore, if nocturnal eating is unavoidable, favor small, protein-rich, low-glycemic items; avoid large, carbohydrate-dense meals during the biological night. It is also suggested to avoid an immediate heavy meal straight off a night shift, preferring sleep first, then a light, phase-appropriate meal upon waking.

Interestingly, recent interventional studies aim to propose sleep and nutritional strategies for night-shift workers. In particular, a controlled trial (ClinicalTrials.gov Identifier: NCT06147089) is evaluating the impact of individualized sleep scheduling and tailored meal timing on metabolic and hormonal outcomes in real-life conditions integrating actigraphy, continuous glucose monitoring, and hormonal and inflammatory biomarkers over a 3-month period with 12-month follow-up [130]. These results are expected to provide evidence-based models for preventive chrononutritional and behavioral strategies aimed at mitigating the metabolic and endocrine disruption induced by night-shift work. Recent data also stressed the behavioral and environmental complexity of circadian disruption in shift work. A study among healthcare shift workers identified recurrent problems in maintaining safe lifestyle habits, including irregular meal timing, meal skipping, limited food accessibility, poor sleep quality, and reduced physical activity [131].

In jet lag, it is suggested to shift meals according to the time of destination, prioritizing a proper local-morning breakfast and avoiding heavy late-evening meals during the first 1–3 nights. As above mentioned, the effects of a KD pattern on the HPA axis and on daily cortisol secretion are conflicting, with opposite results between different animal models and humans. Beyond the benefits proved in terms of cortisol secretion and body composition in obese patients, the effect of VLCKD has also been tested in a group of 15 CD patients, showing a greater improvement of metabolic parameters. After 5 weeks, a significant decrease in BMI, waist circumference, blood pressure, and ACTH levels, as well as an improvement of lipid profile has been obtained, proposing the introduction of KD together with conventional CD therapy to improve metabolic and cardiovascular comorbidities [121]. Therefore, from a clinical point of view, the clinical benefits of KD override the uncertain effects on “biochemical” trends of cortisol’s daily rhythm. Conversely, no data supports the utility of intermittent fasting in hypercortisolism. In humans, prolonged fasting (to 2.5 to 6 days) increases cortisol levels and shifts the peak from the morning to the afternoon. Therefore, data derived from healthy subjects show that intermittent fasting contributes to an increasing level and frequency of cortisol secretion [118].

### 5.2. Pharmacological Modulation on HPA Axis

#### 5.2.1. Drugs Targeting Cortisol Secretion

The use of drugs targeting cortisol secretion and peripheral actions have been approved for the treatment of endogenous CS. Outside this specific indication (pseudo-Cushing, psychiatric disorders, MetS, jet lag/shift work, or complications of exogenous steroids), there is no regulatory approval with the aim of restoring cortisol’s daily rhythm. Regardless the etiology, surgery is the gold standard therapy for CS, aimed to remove the tumor causing the inappropriate secretion of ACTH and, less frequently, cortisol [132]. Medical treatment is used when surgery is not indicated, or in cases of persistent or recurrent hypercortisolism or if prompt control of hypercortisolism is mandatory while awaiting surgery. Steroidogenesis inhibitors such as ketoconazole or metyrapone are effective in reducing mean 24 h cortisol values but they are not useful in completely restoring daily cortisol rhythm, especially in the presence of overt hypercortisolism at baseline [122]. Therefore, there is limited evidence for restoring diurnal cortisol rhythm, at least for standard treatment. Interestingly, Debono demonstrated that the evening administration of metyrapone can restore a physiological cortisol rhythm in MACS, a condition associated with evening and nocturnal increased cortisol exposure. Furthermore, this specific treatment is associated with a reduction in the cardiovascular risk marker IL-6 [133]. The somatostatin analog pasireotide, approved for the treatment of CD, is able to induce a reduction in urinary free cortisol [123]. Despite a reduction in late-night salivary cortisol, suggesting a possible role in restoring cortisol daily rhythm [134], data are conflicting [124]. Real-world data from the ILLUSTRATE study confirmed that osilodrostat, an 11β-hydroxylase inhibitor, led to a marked reduction in urinary free cortisol, which normalized in approximately 70% of patients [135]. An innovative and recent study evaluates the effect of osilodrostat in addressing daily rhythm restoration. In a group of patients stable with twice-daily osilodrostat therapy, the shift to a single equivalent daily dose administered at the evening, improves daily cortisol profiles, quality of life, and sleep [125], suggesting a possible role of this therapeutic scheme in improving the daily cortisol profile.

#### 5.2.2. Drugs Acting on Metabolic Complications and Supplements

Drugs acting on metabolic complications are often used for patients with CS and MetS, as well as in patients with other causes of altered cortisol secretion, in the presence of metabolic diseases. Clinical evidence regarding their specific effects on cortisol’s daily rhythm is not well known and derives from animal models. Metformin, the first line treatment for type 2 diabetes, targets the circadian gene PRKAB1, which encodes for a regulatory subunit of the AMP-activated protein kinase (AMPK), a master regulator of energy metabolism. Hypercortisolism inhibits AMPK activity. Metformin promotes increased leptin and decreased glucagon levels, leading to AMPK activation, which in turn resulted in the inhibition of acetyl CoA carboxylase, the rate-limiting enzyme in fatty acid synthesis. Furthermore, metformin leads to the activation of liver casein kinase I α and muscle casein kinase Iε, which in turn modulates the positive loop of the circadian clock [126]. Considering the AMPK–clock crosstalk observed in preclinical and in vitro models, it is biologically plausible that evening modified-release metformin frequently used in clinical practice, might favorably modulate peripheral clock timing and, indirectly, diurnal cortisol dynamics. However, no human randomized trials have tested this hypothesis yet. Therefore, robust data confirming the possible role of metformin in restoring cortisol’s daily rhythm is lacking.

The hormone melatonin is produced by the pineal gland. Its production begins at around 22:00–23:00 h, with a peak at 2–3 a.m., and lowest level at 9–10 a.m. [136]. Production is regulated by light exposure, while it is not affected by physical activity, sleep, meals, stress, or the menstrual cycle, being independent from pathways involving GC action [13].

Melatonin treatment effects have been reported in several disorders such as certain tumors, cardiovascular diseases, and psychiatric disorders, but its use is well known in the treatment of sleep disorders and to prevent jet lag [137]. Clinically, timed dosing (typically 0.5–5 mg at local bedtime) reduces jet-lag symptoms after eastward travel across ≥ five time zones. Its hypnotic effect is modest and without risk of dependence, offering a valid option for rhythm disorders with comorbid insomnia or mood symptoms. Concerning cortisol rhythm, beyond the effects on central clock, the adrenal cortex also expresses melatonin receptors. In has been demonstrated that melatonin can modulate adrenal clockwork and steroidogenesis by blunting ACTH-induced PER1, steroidogenic acute regulatory protein (StAR), and 3β-Hydroxysteroid dehydrogenase (3β-HSD) expression, modulating adrenal responsiveness to hypothalamic–pituitary regulation [138]. However, despite these findings, the relationship between melatonin and cortisol secretion modulation is more complex and data remains limited. Some authors have demonstrated that melatonin improves sleep quality and reduces night salivary cortisol is specific clinical settings [139]; conversely, other studies found a generic increase in salivary cortisol levels in healthy volunteers [140].

Despite these limitations, from a practical point of view, the combined central and adrenal mechanisms, together with consistent benefits in jet lag and shift-work contexts, support melatonin as a pragmatic adjunct to disease-directed care and chrononutritional measures for possible rhythm restoration.

Finally, probiotics and short-chain fatty acid-promoting diets (fermented foods and fiber) show small but reproducible dampening of stress-related cortisol responses and can be used as safe adjuvants within a Mediterranean framework; omega-3 fatty acids and vitamin C have supportive evidence for reducing HPA axis reactivity in selected settings.

## 6. Conclusions

The mechanisms underlying the condition of “inappropriate cortisol secretion” represent one of the most fascinating topics in clinical practice. Recent evidence suggests that the well-known health outcomes related to hypercortisolism depend less on the absolute amount of cortisol but rather on the disruption of its daily profile, characterized by a clear morning rise and a low evening nadir. The alteration of cortisol’s daily rhythm represents a paradigmatic example of misalignment between the central clock and peripheral tissues. These alterations can be detected not only in CS, but also in other conditions characterized by functional hypercortisolism, representing a cause of increased metabolic and neurobehavioral risk. It is therefore not a surprise that current research centers on this question. Alongside disease-specific treatments when indicated, personalized nutritional suggestions (chrononutrion and meal composition) offer practical and useful support to improve the possibility of cortisol rhythm realignment. Chronobiotic support (melatonin and other supplements) can help the consolidate phase in selected contexts, while standard metabolic therapies improve metabolic parameters, despite that their effects on direct cortisol secretion have to be elucidated. Moving forward, progress will depend on clarifying the pathophysiological mechanisms and implementing corrective strategies grounded in lifestyle, especially in nutrition, as well as pharmacology. Furthermore, considering the conflicting data, future studies should prioritize randomized clinical trials directly aimed to test the effects of a specific dietary pattern, as well as drugs used to control hypercortisolism, on restoring—at least partially—the normal cortisol daily rhythm. A close multidisciplinary collaboration between physicians and nutritionists is mandatory.

## Figures and Tables

**Table 1:** Suggestions to restore cortisol daily rhythm (MetS: metabolic syndrome; BMI: body mass index; CS: Cushing syndrome; MACS: mild autonomous cortisol secretion; CD: Cushing disease; QoL: quality of life).

Strategy	Specific Intervention	Rationale	Clinical Evidence
Chrononutrition	Early time-restricted eating, avoiding late-night meals	Aligns feeding cycles with central pacemakers, improves glycemic control, reduces evening cortisol elevations	Beneficial in healthy subjects and MetS; BMI reduction [115,117,118]
	Low-glycemic, low-fat meals in hypercortisolism	Lower postprandial cortisol excursion	Clinical rationale in CS and MACS [119]
	Shift workers: avoid food intake between midnight–6 a.m., prefer protein-rich light meals if eating at night	Reduces daily misalignment, improves alertness/satiety	Observational data [117]
	Jet lag: adapt meal timing to destination (prioritize breakfast, avoid late evening meals)	Improves daily realignment	Practical clinical advice [120]
	Ketogenic diet (KD/VLCKD)	Improves body composition and metabolic parameters	Evidence in obese and CD patients; improves metabolic comorbidities [121]
Pharmacological (only in CS)	Ketoconazole, metyrapone	Inhibit steroidogenesis; lowers mean cortisol	Does not fully restore rhythm; evening metyrapone may help in MACS [122]
	Pasireotide	Reduces ACTH and late-night cortisol	Conflicting data [123,124]
	Osilodrostat (evening dosing)	Inhibits steroidogenesis; improves daily profile, QoL, sleep	Promising evidence in CD patient [125]
Pharmacological (metabolic/supplements)	Metformin (evening modified release)	Modulates peripheral clocks	Preclinical evidence; not yet confirmed in human trials [126]
	Melatonin (0.5–5 mg at bedtime)	Modulates central and adrenal clocks, blunts ACTH-induced steroidogenesis	Effective in jet lag [127]
	Probiotics, SCFA-promoting diets, Mediterranean diet, Omega-3 fatty acids, and vitamin C	Reduce stress-related cortisol responses and HPA reactivity in selected contexts	Supportive but modest evidence [70,71,75,76]

## Data Availability

No new data were created or analyzed in this study. Data sharing is not applicable to this article.
